# Comparing motivational, self-regulatory and habitual processes in a computer-tailored physical activity intervention in hospital employees - protocol for the *PATHS* randomised controlled trial

**DOI:** 10.1186/s12889-017-4415-4

**Published:** 2017-05-26

**Authors:** Dominika Kwasnicka, Corneel Vandelanotte, Amanda Rebar, Benjamin Gardner, Camille Short, Mitch Duncan, Dawn Crook, Martin S. Hagger

**Affiliations:** 1 Health Psychology & Behavioural Medicine, School of Psychology and Speech Pathology, Faculty of Health Sciences, Curtin University, Perth, Australia; 2 Physical Activity Research Group, School of Health, Medical and Applied Sciences, Central Queensland University, Rockhampton, QLD Australia; 3 Department of Psychology, Institute of Psychiatry, Psychology & Neuroscience (IoPPN), King’s College London, London, UK; 4 Freemasons Foundation Centre for Men’s Health, South Australian Health and Medical Research Institute, Faculty of Health Sciences, The University of Adelaide, Adelaide, Australia; 5 Priority Research Centre for Physical Activity and Nutrition, Faculty of Health and Medicine, School of Medicine & Public Health, The University of Newcastle, Callaghan, Australia; 6 St John of God Subiaco Hospital, Perth, Australia; 7 Faculty of Sport and Health Sciences, University of Jyväkylä, Jyväkylä, Finland

**Keywords:** Computer-tailoring, Behaviour change, Behaviour maintenance, Habit, Healthcare professionals, Physical activity, Web-based, Randomised controlled trial

## Abstract

**Background:**

Most people do not engage in sufficient physical activity to confer health benefits and to reduce risk of chronic disease. Healthcare professionals frequently provide guidance on physical activity, but often do not meet guideline levels of physical activity themselves. The main objective of this study is to develop and test the efficacy of a tailored intervention to increase healthcare professionals’ physical activity participation and quality of life, and to reduce work-related stress and absenteeism. This is the first study to compare the additive effects of three forms of a tailored intervention using different techniques from behavioural theory, which differ according to their focus on motivational, self-regulatory and/or habitual processes.

**Methods/Design:**

Healthcare professionals (*N* = 192) will be recruited from four hospitals in Perth, Western Australia, via email lists, leaflets, and posters to participate in the four group randomised controlled trial. Participants will be randomised to one of four conditions: (1) education only (non-tailored information only), (2) education plus intervention components to enhance motivation, (3) education plus components to enhance motivation and self-regulation, and (4) education plus components to enhance motivation, self-regulation and habit formation. All intervention groups will receive a computer-tailored intervention administered via a web-based platform and will receive supporting text-messages containing tailored information, prompts and feedback relevant to each condition. All outcomes will be assessed at baseline, and at 3-month follow-up. The primary outcome assessed in this study is physical activity measured using activity monitors. Secondary outcomes include: quality of life, stress, anxiety, sleep, and absenteeism. Website engagement, retention, preferences and intervention fidelity will also be evaluated as well as potential mediators and moderators of intervention effect.

**Discussion:**

This is the first study to examine a tailored, technology-supported intervention aiming to increase physical activity in healthcare professionals. The study will evaluate whether including additional theory-based behaviour change techniques aimed at promoting motivation, self-regulation and habit will lead to increased physical activity participation relative to information alone. The online platform developed in this study has potential to deliver efficient, scalable and personally-relevant intervention that can be translated to other occupational settings.

**Trial registration:**

Australian New-Zealand Clinical Trial Registry: ACTRN12616000462482, submitted 29/03/2016, prospectively registered 8/04/2016.

## Background

Physical inactivity is related to increased risk of a number of chronic diseases (e.g., cardiovascular disease, Type 2 diabetes, certain types of cancer, obesity) [1, 2]. Engaging in regular physical activity is related to reduced risk of chronic diseases [2]. In addition, positive outcomes of physical activity include improved quality of life, better sleep, and reduced stress [3, 4]. World Health Organisation physical activity guidelines for adults to gain health benefits is to undertake at least 150 min of moderate intensity physical activity per week (e.g., walking, cycling), or at least 75 min of vigorous intensity physical activity per week (e.g., running, playing football) [5]. Globally, 1 in 4 adults does not meet these recommendations [5].

Healthcare professionals have a key role to play in the promotion of physical activity [6]; however they often do not meet guideline levels of physical activity themselves. For instance a study focussing on nurses’ physical activity (*N* = 325) indicated that more than half of the assessed sample did not meet guideline levels of physical activity [7]. Those who were less active were also more likely to report poor general health and worse sleep patterns than their active counterparts. Despite significant health education among health care professionals, it appears that the health knowledge often does not translate into their own health behaviours [7–9].

In addition, there is a relationship between personal physical activity behaviours of healthcare professionals and their health-promotion practice. A systematic review [10] of cross-sectional studies (*N* = 13) investigating this relationship found that a higher personal physical activity level in healthcare professionals was associated with higher physical activity-promoting practices in most studies. Health professionals with positive attitudes towards physical activity were also more likely to promote physical activity to their patients.

Healthcare professionals often exhibit unhealthy lifestyle behaviours with work-related stress identified as the most frequently-cited reason [11]. Professionals report that hospitals are a highly stressful work environment, and irregular shift work often places an additional strain on the hospital employees [12]. Sleep patterns and sleep quality among healthcare professionals were insufficient for good health [13] and physical activity is a recognised means to improve sleep quality [14]. In addition, nurses are prone to suffer lower back pain [15] and physical activity is recommended to this occupational group for managing back pain. As such, there is strong rationale for promoting physical activity among healthcare professionals.

The workplace is reported as a suitable environment for making changes in the physical activity and improving health of employees [16]. Increased participation in physical activity in healthcare professionals can be promoted using behavioural interventions which utilise persuasive strategies and techniques to encourage individuals to change their behaviour (e.g., goal setting, planning, providing social support) [17, 18]. Such interventions have been shown to be effective in promoting increased uptake and maintenance of physical activity, including in the workplace [19–21], hence they may be suitable for promoting physical activity to healthcare professionals.

Other than identifying the importance of context in the delivery of interventions, such as the occupational setting for healthcare workers, it is also important to look at the means by which interventions may be delivered to employees in this context [22]. Recently computer-tailoring has been used to deliver behavioural interventions in health contexts and it has received increased attention as a means to effectively deliver personalised interventions to a wide audience at a relatively low cost [23]. These interventions provide users with individualised feedback based on their demographic profile and preferences. Users are prompted to provide information salient to the intervention (using an online questionnaire) and are subsequently provided with tailored feedback including information on behaviour change matched to their requirements and consistent with their responses. Pre-defined algorithms generate tailored content for the user based on user-provided information and the relevant behaviour change content that is stored in a database including all possible response options [24].

Web-based interventions have a number of advantages over face-to-face interventions: they have wide reach, comparatively low cost of implementation and delivery, and flexibility of intervention use at times and location convenient for the user [25, 26]. Tailoring web-based interventions also carries distinct advantages over non-tailored approaches: participants are presented only with relevant and personalised information, and non-relevant information can be omitted. This in turn may increase engagement and persuasion [27, 28]. In computer-tailored interventions, less information is presented to the users and more attention is directed to the relevant intervention content. In addition, consistent with the elaboration likelihood model of persuasion [29], information in the intervention is more likely to be thoughtfully attended to when it is personally relevant and when readers are motivated. Therefore, tailored interventions are more likely to be attended to and should lead to longer lasting behavioural change.

In addition to computer tailoring, there is also a growing literature supporting the use of short message service (SMS) or ‘text’ messaging to promote health behaviour. Research has demonstrated that delivering health behaviour messages via text messaging can promote health behaviour change in numerous contexts, including physical activity [30]. Text messaging has also been used in conjunction with web-based interventions to improve health behaviour and to enhance their effectiveness. A systematic review reported that the effectiveness of web-based interventions can be enhanced by the inclusion of SMS as a means to remind participants of intervention content or to augment it [31]. Therefore, adoption of web-based tailored interventions to promote physical activity augmented with text messaging, may be a useful, cost-effective means to promote physical activity to healthcare professionals.

In the context of physical activity promotion, computer-tailored behavioural interventions have been reported as effective [26, 32]; however further investigation of the mechanisms is needed in terms of evaluating these interventions and their effectiveness across multiple contexts. Investigating the mechanisms that can improve the effectiveness of computer-tailored interventions is crucial. Therefore, studies that isolate specific behaviour change techniques (BCTs) within intervention conditions using factorial designs will provide robust evidence for testing the impact of specific BCTs [18]. This means that knowledge of the specific individual components that are most likely to change behaviour will be identified.

### Theories of behaviour change and maintenance

Health behaviour change interventions are usually underpinned by psychological theories of behaviour change and maintenance. Many such theories have focused on *motivation* and intentions to explain and change behaviour (e.g., the Theory of Planned Behaviour) [33]. These theories view lack of engagement in health behaviours (e.g., physical activity) as primarily a problem with motivation, such that increasing motivation will directly lead to increases in behavioural participation. The theories also assume that motivation is a function of an individuals’ explicit, consciously-held beliefs about the behaviour [33]. However, theories of *volition* [34] indicate that motivation is a necessary but insufficient condition for behavioural enactment and suggests that volitional strategies (e.g., planning) that operate in a post-decisional manner (i.e. after intentions have been formed) lead to the effective execution of intentions into action because they enable an individual to more effectively recall their intended behaviour via prompts or cues that have been linked to the desired behaviour [35].

Dual process theories offer a broader perspective that integrates *motivational* and *volitional* processes, but also recognises that behaviour may also be influenced by more implicit, non-conscious processes [36–39]. Dual process theories propose that health behaviours are enacted through two processes – the conscious, deliberative processes such as those described in motivational and volitional theories, as well as non-conscious processes that occur automatically, outside an individual’s deliberation. The implicit processes reflect initiating or engaging in actions with very little deliberative, reasoned, and conscious decision making processes outlined in traditional motivational theories. Behaviours that may initially be controlled through deliberative, motivational pathways behaviours can, through a *habit-formation process* become controlled by automatic processes [40]. Habit formation likely occurs through learning of context-behaviour associations via context-dependent repetition of the behaviour usually in the presence of some rewarding contingency [41]. In some cases, the behaviour is enacted because repetition and experience has led the behaviour to occur without the need for any conscious control, as such the behaviour is classed as ‘habitual’ [42, 43].

Researchers applying these theories to predict and change behaviour have identified strategies and techniques to promote the development of habits [40]. These techniques should be separable and able to be isolated from techniques that promote, for example, increased behavioural engagement via the motivational and volitional pathways identified in dual process theories. As a consequence, factorial-type designs should be able to demonstrate the unique effects of techniques related to motivation and volition and techniques related to habit promotion on health behavior change. Such designs may also permit demonstration of whether the addition of components targeting specific processes (e.g., motivational, volitional, habitual) may lead to incremental changes in behaviour relative to each set of techniques alone.

### The present study

There is a need for cost-effective, efficacious behavioural interventions to increase physical activity in healthcare professionals [13]. Research has suggested that theory-based interventions adopting multiple BCTs are effective in increasing physical activity behaviour [31]. Recent research efforts have focused on linking BCTs with underlying theoretical mechanisms of action [44]. However, few studies have systematically examined the efficacy of specific groups of BCTs that are thought to operate through one of the three specified processes (motivational, volitional, and habit-forming) and assessed their independent and additive effects on behaviour change. The purpose of the current study, the Physical Activity Tailored intervention in Hospital Staff (*PATHS*) study, is to evaluate the efficacy of a computer-tailored intervention to increase physical activity and quality of life, and decrease work-related stress and absenteeism, in hospital healthcare workers. The intervention will examine the additive effects of groups of BCTs derived from theories of motivation, volition, and habit on physical activity behavior change. The research is unique as it will provide the first evidence of the efficacy of techniques derived from these three theoretical approaches applied in a workplace context. The study will adopt a randomised controlled design to test and compare the effects of three distinct groups of BCTs derived from theory and focusing different processes of behaviour changes on study outcomes: motivation, self-regulation, and habit formation.

Specifically, the intervention will include motivational techniques to increase motivation and self-efficacy to engage in physical activity, self-regulatory techniques to increase capability and skills to initiate and regulate behaviour change, and habit-based techniques to increase the automatic, non-conscious tendencies to engage in physical activity on presentation of contextual and time-relevant cues. Three intervention conditions are proposed, each defined by the sets of BCTs in an additive design: (1) motivational BCTs; (2) motivational plus self-regulatory BCTs; and (3) motivational plus self-regulatory and habit formation BCTs. The intervention groups will be compared to a control condition that will receive basic non-tailored information about physical activity.

## Methods

### Participants

Full- and part-time hospital staff including midwives, clinical nurse managers, clinical nurses, registered nurses, enrolled nurses, and patient care assistants will be eligible to participate in the *PATHS* study. Participants will be recruited from hospitals in Perth, Western Australia. Staff working in shift and non-shift patterns will be eligible to participate. No restrictions will be placed on the type of contract and number of hours worked. Participants are eligible if they fail to meet the recommended weekly level of 150 min of moderate intensity physical activity because the intervention is designed for people who fail to meet this level.

Individuals who self-report a physical condition or impairment preventing them from being physically active will be excluded from the study. They will be screened using the Physical Activity Readiness Questionnaire (PAR-Q) prior to study consent [45]. However, individuals who are not eligible to participate in the study based on PAR-Q measures can still consult their GP to obtain approval. Hospital employees who do not have a mobile phone that allows them to receive text messages, who do not have access to internet on their mobile phone, computer or tablet outside of work, who are already meeting weekly recommended levels of physical activity or are currently participating in another physical activity program (e.g., a structured weight loss program, regular meetings with personal trainer or coach) will be excluded.

### Recruitment

Participants will be recruited from different hospital wards via email messages and printed letters that will be sent to all potentially eligible employees, accompanied by a description of the study and its requirements and inviting them to take part. Study posters and leaflets advertising the study will be placed throughout hospital premises. Members of the *PATHS* team will also visit each ward to promote the study, alongside ward managers. The promotional materials will advertise the study as research investigating the development of a web-based tool to improve employees’ physical activity, wellbeing and health. Recruitment materials (including posters, leaflets, emails, and printed letters) will direct interested staff to the project website, which will provide study information and will allow them to assess eligibility and register to participate. A formal statement of consent will be signed in person at the first meeting with the study researcher.

### Study design

**Fig. 1:**
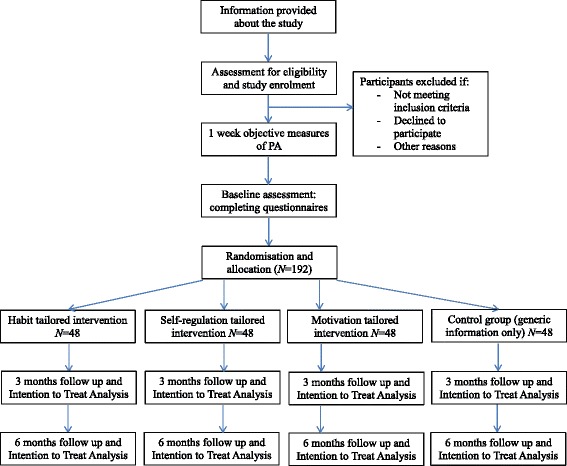
CONSORT flow diagram [91]

The *PATHS* study is a pragmatic four-group intervention trial adopting a randomised control design with assessments of primary and secondary outcomes at baseline and 3-month follow-up time points (see Fig. 1). We chose randomisation of individuals within hospitals (rather than randomisation of hospitals or wards) because individual randomisation was more efficient and more feasible with continuous recruitment of the participants; program was delivered to individuals rather than groups so contamination across intervention groups was not considered a major risk [46]. The project comprises three intervention conditions and a no intervention condition, in which participants receive information about the study only. This will serve as a control condition. Each condition includes a group of theory-based BCTs designed to test the additive effects of the specific BCT groups on study outcomes:

1. **Control condition** – provision of information about physical activity only
2. **Motivation condition** – provision of information + motivational BCTs
3. **Self-regulation condition** – provision of information + motivational BCTs + self-regulatory BCTs
4. **Habit condition** – provision of information + motivational BCTs + self-regulatory BCTs + habit formation BCTs

### Procedure

After screening, eligible participants will be contacted by a study researcher who will arrange a one-on-one meeting (Figure 1, CONSORT flow diagram). During the meeting, the participant will receive a detailed explanation of the study, and its requirements and expectations from the researcher. They will also be provided with an information sheet providing the same information and outlining their rights to confidentiality and to withdraw from the study at any stage without consequence. Once the participant has had the opportunity to confirm their understanding of the project and have any questions answered, they will be asked to sign an informed consent form.

A study researcher will set up and initiate a GENEActiv activity monitor [47] for each participant, including programming the participant’s code number, date of birth, height, and handedness. Each monitor will be programmed to start collecting data from set up. Study participants will be required to wear the monitor on their non-dominant upper arm and will be provided with detailed instructions on how to wear the device. Participants will be instructed to wear it continuously throughout day and night for one week after the first meeting.

Eight days later, participants will meet with the researcher again to return the activity monitor. The study facilitator will check if sufficient data have been collected by the device (i.e., at least five days of wear with a minimum 16 h wear per day) [48]. If the participant fails to wear the activity monitor for the minimum time specified, they will be asked to wear it again for another seven days. At this second meeting, participants will also be asked to complete web-based baseline questionnaires using a provided tablet (Time 1).

After the second meeting, participants will be emailed a personalised login/password, and link to the website so they are able to login to the web-based *PATHS* study platform used to deliver the tailored intervention messages. Participants will be automatically randomly allocated online in an equal ratio to one of the four possible study conditions; a block randomisation will be used with block size of 16. If allocated to one of the three intervention conditions, they will be able to complete the first intervention session on the website immediately after completing the baseline assessments. If allocated to the control condition, they will receive an email directing them to the *Library* site for physical activity relevant information. The *Library* is a sub-section of the website with non-tailored evidence-based information about physical activity,

Across all conditions, participants will be encouraged to use and interact with the web-based *PATHS* platform for three consecutive months, completing sessions every fortnight. At the end of month three, participants will meet with the researcher and will be provided with a GENEActiv activity monitor to be worn for one week immediately post-intervention to assess physical activity. Participants will meet with the researcher again one week after they received the activity monitor to complete the post-intervention questionnaires and return the activity monitor (Time 2). All researchers collecting the data will be blinded to the allocation of participants’ study condition.

Following the three-month period, participants will be thanked for their study contribution via personal email and text message, and their names will be automatically entered into a prize draw allowing them to win shopping vouchers for their participation (four shopping vouchers, 20 AUD value per each hospital). Participants allocated to the control condition will then be given the opportunity to receive the content from one of the intervention conditions so that they are not prevented from participating in a potentially effective intervention. They will receive login details, password and link to the web-based tailored platform providing them with the option to engage in one of the three intervention conditions, chosen randomly. All study participants will be told that there are four different versions of the same program that differ in content and behaviour change techniques used. Participants will be asked not to share or compare intervention content to avoid study contamination among participants working on the same ward. They will be asked in the post intervention evaluation if they adhered to the aforementioned recommendation.

### Intervention content

#### Control condition

Participants assigned to the control condition will be provided with information about physical activity recommendations compiled in the *Library* and will have the opportunity to rate the content (ranging from 0 stars [*not interesting and not useful*] to 5 stars [*very interesting and very useful*]). The presentation order of the articles will move up or down on the list based on the ratings given by the platform users, with the ones rated the highest displayed at the top of the page. The information will be factual and non-personalised. BCTs used in the control group are: providing information about health consequences [49], providing information about social [50], environmental [51] and emotional consequences [52]. Websites that provide information only have not been shown to have strong effects on health behaviour change [27] and is effective as a control group in the current study as it provides a control for information load and contact with the study team.

#### Motivation condition

Participants assigned to the motivation condition will receive personalised messages based on BCTs to promote intention and self-efficacy to engage in physical activity [53]. Specifically, participants will be provided with advice on how to work towards their goals and to maintain motivation in the face of barriers [54, 55].

#### Self-regulation condition

Participants assigned to the self-regulation condition will receive the content from the motivation condition along with content promoting self-regulation. Specifically, participants will be encouraged to set outcome-specific goals [56] and to self-monitor their progress towards these goals by monitoring how much time they spent doing physical activity. They will also be prompted to form action and coping plans [57], specifying when, where and how they will perform their physical activities, and identifying barriers to their physical activity participation and how they might overcome them. Participants will be encouraged to specifically focus on self-monitoring in high risk situations [58], such as when they are tired or stressed and their capacity for self-regulation is most likely to be compromised [59, 60]. Participants will also receive information on managing multiple goals [61] and self-regulating the situation when competing activities arise (e.g., the desire to be active and healthy conflicts with other situational options such as watching TV and resting).

#### Habit condition

Participants assigned to the habit condition will receive the content from the motivation and self-regulation conditions along with content promoting habit development (e.g., recognising prompts to action and learning to act upon these prompts). Participants will receive information relevant to implementation intentions with context-specific prompts [62] (e.g., forming simple prompt-dependent plans to engage in physical activity when the prompt occurs). They will be asked to recognise and identify situational, contextual and time-based cues that can prompt them to be more active. Participants will be encouraged to develop a habit of instigating physical activity without having to consciously remember [63]. In order to do things automatically behavioural repetition of the same activity in the same contexts will be encouraged; participants will have the choice of which context they wish to do the activity.

### Intervention delivery

**Fig. 2:**
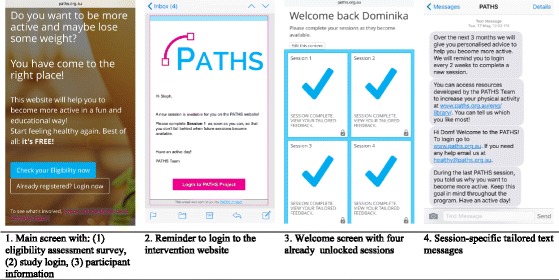
*PATHS* study – intervention screenshots – content examples

The intervention will be delivered via the *PATHS* website [64] (see Fig. 2 for screenshot examples). Participants in all conditions will log-in to the web-based intervention using their email address and password created while going through the eligibility assessment process. Participants in all conditions will have access to the *Library* in the form of brief clear-language descriptions of physical activity recommendations and information on the importance of physical activity. Given research demonstrating that providing information about health behaviour alone is not sufficient to change behaviour [65], but also recognising the need to provide a rationale for engaging in the website and to ensure that information is controlled across conditions, all study participants will have access to the *Library*.

All participants will also have access to *Frequently Asked Questions* page, a sub-section of the website about practicalities of its use (e.g., how to change their password, basic features, tips on navigating through the website). This sub-section of the website has been designed based on piloting previous similar website [66]. Participants will be able to contact an intervention facilitator if they have any additional questions about participating in the study. There will also be an *About* page available – a sub-section of the website that will (1) explain the reasons why the intervention was developed and (2) introduce the *PATHS* study research team to add credibility. A *Frequently Asked Questions* page and an *About* page, also developed from previous investigations piloting the platform, will be slightly different for each intervention condition to account for the differences between conditions.

Participants allocated to the three intervention groups will have access to additional intervention features. Each fortnight, they will be prompted via email to access the website and to answer short online surveys and receive tailored feedback based on the information provided in their responses. Depending on their group allocation (Tables 1 and 2), participants will receive feedback aimed at promoting motivation (motivation group), motivation and self-regulation (self-regulation group), or motivation, self-regulation and prompts for actions (habit group). Information pertinent to the participant’s condition will be delivered via personally-tailored messages presented as text on a webpage accompanied by appropriate pictures and graphs with tailored information (e.g., graphs showing participant’s change in physical activity or weight throughout the intervention). The intervention will include six consecutive sessions that will be presented fortnightly for three consecutive months.

**Table 1:** Intervention content for *PATHS* study **-** overview

Session number	Motivation condition	Motivation + self-regulation condition	Motivation + self-regulation + habit condition
1	Motivation and self-efficacy	Motivation, self-efficacy and self-monitoring	Motivation, self-efficacy, self-monitoring and habit development
2	Goal setting	Goal setting and action planning	Goal setting, action planning and forming positive habits
3	Self-efficacy, barriers identification and staying motivated when facing barriers	Self-efficacy, barrier identification and staying motivated and self-regulating when facing barriers	Self-efficacy, barriers identification and staying motivated, self-regulating in face of barriers and recognising cues to action
4	Social support and motivation	Social support and motivation, and self-monitoring with others	Social support and motivation, self-monitoring and forming activity routines with others
5	Experiencing barriers to being active and staying motivated	Experiencing barriers to being active and staying motivated; relapse prevention	Experiencing barriers to being active and staying motivated; reasons for falling back into bad habits; relapse prevention
6	Summary: staying motivated	Summary: staying motivated and self-regulating	Summary: staying motivated, self-regulating and maintaining habits

**Table 2:** Variables assessed, feedback topics, BCTs and constructs targeted in each session (Motivation + Self-regulation + Habit condition combined)

Session number	Session topic	Variables assessed (number of questions)	Feedback topics (number of permutations, i.e. possible variations of the response)	BCTs included^a^	Constructs targeted
1	Motivation, self-efficacy, self-monitoring and habit development	- Demographics (5) - Physical activity (10) - PA goal (1; 5 options) - PA self-efficacy (1; Not at all certain/ Moderately certain / Highly certain) - Preference for PA routine (1; 5 options) - Habit Automaticity Index (4 questions) - Feedback on the session after tailored advice (1)	- Introduction based on the condition (3) - Motivation: Physical activity guideline according to the selected main motivation (7) - Self-regulation: Feedback on current physical activity level in relation to the main motivation chosen, presented with graph (87) - Habit: Developing habits based on preferred PA, need for behavioural repetition (5) - Habit: Recognising prompts in relation to the habit automaticity level (3) - Self-regulation: Setting a goal for the next two weeks based on self-efficacy levels; goal is predetermined (9) - Conclusion based on the condition and main motivation (15)	1.1. Goal setting (behaviour) 2.2. Feedback on behaviour 5.1. Information about health consequences 5.6. Information about emotional consequences 7.1. Prompts/cues 8.3. Habit formation 15.1. Verbal persuasion about capability	Knowledge, goals, intrinsic motivation, autonomy, self-efficacy, intentions, outcome expectations, cues and habits
2	Goal setting, action planning and forming positive habits	- Physical activity (10) - Weight (1) - Goal setting (2) - Open questions about long and short term goals (2) - Habits and prompts (2) - Action planning phrased with/without references to habit – open answers (5) - Feedback on the session after tailored advice (1)	- Welcome based on the condition (3) - Self-regulation: PA guidance refresher based on the main goal (5) - Physical activity progress feedback (27) - Motivation: Long and short term goals with SMART goals principles (4) - Habit: Feedback on developing habits and noticing prompts (4) - Self-regulation: Action plan (1) - Habit: Action plan with reference to behaviour repetition in a stable context (1) - Conclusion based on the condition (3)	1.1 Goal setting (behaviour) 1.3. Goal setting (outcome) 1.4. Action planning 2.2. Feedback on behaviour 7.1. Prompts/cues 8.3. Habit formation	Knowledge, self-efficacy, attitudes, perceived behavioural control, goals, intentions, cues and habits
3	Self-efficacy, barriers identification and staying motivated, self-regulating in face of barriers and recognising cues to action	- Physical activity (10) - Weight (1) - Coping self-efficacy (6) - Action plan completed (1) - Main prompts (1; 5 options) - Action plan evaluated open answers (5) - Confidence to recognise prompts (1) - Feedback on the session after tailored advice (1)	- Welcome based on the condition and prompts recognised for Habit group (9) - Motivation: Boosting your confidence and staying motivated based on identified barriers (6) - Self-regulation: Physical activity progress feedback includes graphs (52) - Habit: Feedback on developing habits (6) - Habit: Noticing prompts (5) - Self-regulation: Action plan (1) - Habit: Action plan with reference to behaviour repetition in a stable context (1) - Conclusion based on the condition (3)	1.4. Action planning 1.5. Review behaviour goal(s) 1.6. Discrepancy between current behaviour and goal 5.4. Monitoring of emotional consequences^b^ 5.5. Anticipated regret 5.6. Information about emotional consequences 7.1. Prompts/cues 8.3. Habit formation	Knowledge, self-efficacy, attitudes, perceived behavioural control, coping self-efficacy, goals, intentions, emotions, cues and habits
4	Social support and motivation, self-monitoring and forming activity routines with others	- Physical activity (10) - Weight (1) - Positive social support (3) - Negative social support (3) - Influence of others (1) - Feedback on the session after tailored advice (1)	- Welcome based on the condition (3) - Self-regulation: Physical activity progress feedback (52) - Motivation: Positive influence of others and dyadic plans (20) - Self-regulation: Positive influence of others and dyadic plans (20) - Habit: Positive influence of others and dyadic routines (20) - Negative influence of others and staying motivated when others are not supportive (20) - Encouraging other people to be active (2) - Conclusion based on the condition (3)	1.5. Review behaviour goal(s) 1.6. Discrepancy between current behaviour and goal 1.7. Review outcome goal(s) 3.2. Social support (practical) 3.3. Social support (emotional) 6.3. Information about others’ approval 10.9. Self-reward 12.2. Restructuring the social environment	Self-efficacy, knowledge, goals, intrinsic motivation, autonomy, social support, subjective norm, habit, routine
5	Experiencing barriers to being active and staying motivated; reasons for falling back into bad habits; relapse prevention	- Physical activity (10) - Weight (1) - Behavioural barriers (2) - Open question re-evaluating short term goal for PATHS (1) - Habit development (2) - Feedback on the session after tailored advice (1)	- Welcome based on the condition (3) - Self-regulation: Physical activity progress feedback (52) - Habit: Maintaining positive habits – coping planning (4) - Motivation: Relapse prevention based on the main barrier selected with an emphasis on staying motivated (10) - Self-regulation: Relapse prevention based on the main barrier selected with an emphasis on staying motivated and self-regulating (10) - Habit: Relapse prevention based on the main barrier selected with an emphasis on staying motivated/self-regulating and maintaining positive habits (10) - Conclusion based on the condition (3)	1.2. Problem solving 1.7. Review outcome goal(s) 7.3. Reduce prompts/cues 8.2. Behaviour substitution 8.4. Habit reversal 11.2. Reducing negative emotions 12.3. Avoidance/ reducing exposure to cues for the behaviour	Self-efficacy, intrinsic motivation, goals, intentions, knowledge, attitudes, perceived behavioural control, emotions, self-regulation, habits, cues-to-action, behavioural barriers
6	Summary: staying motivated, self-regulating and maintaining habits	- Physical activity (10) - Weight (1) - Habit Automaticity Index (4 questions) - Feedback on the session after tailored advice (1)	- Welcome based on the condition (3) - Self-regulation: Physical activity progress feedback with graph (52) - Self-regulation: Weight changes throughout the program with graph (16) - Motivation: Tips to stay motivated (1) - Self-regulation: Tips to stay motivated and to self-regulate PA (1) - Habit: Tips to stay motivated and to self-regulate PA and to follow newly developed routines (1) - Motivation: Success stories from other hospital employees who increased their PA (1) - Conclusion based on the condition (3)	2.2. Feedback on behaviour 2.7. Feedback on outcome (s) of behaviour 5.1. Information about health consequences 5.6. Information about emotional consequences 6.3. Information about others’ approval 8.1. Behavioural practice/rehearsal 9.2. Pros and cons	Self-efficacy, Intrinsic motivation, Autonomous Motivation, Intention to maintain the behaviour, Habit

^a^ Names and numbers according to V1 Behaviour Change Taxonomy: Michie, Susan, et al. “The behavior change technique taxonomy (v1) of 93 hierarchically clustered techniques: building an international consensus for the reporting of behavior change interventions.” Annals of behavioral medicine 46.1 (2013): 81–95.

^b^ Use of this BCT will depend on the option selected by the participants.

Numbers in brackets indicate the possible number of feedback permutations

Each fortnight, participants will be provided with a new session including a recap of the messages from the previous two weeks along with new personally-relevant content and tailored feedback. If participants do not complete the session on time, they will be able to do so when they next login. Each session will end with a pop-up notification asking users to use a star rating system (*What did you think of this feedback?* 0 to 5 stars) and a qualitative *feedback box* to receive open-ended comments relating to each session content (*Why did you rate it this way?* [optional]).

During each intervention session, participants will receive personally-relevant content incorporating BCTs relevant to their allocated intervention condition (Table 2). To generate the content-specific information, participants will be asked about their physical activity during the previous week at the beginning of each intervention session (including their low, moderate and high intensity physical activity, and resistance training, specifying number and length of sessions). They will also be asked questions regarding the psychological determinants of physical activity, physical resources, and social and environmental factors relevant to the condition to which they are allocated. For example, during the session on goal setting, participants allocated to the Motivation condition will receive information which prompts them to form activity goals and to stay motivated to achieve them. Participants allocated to the Self-regulation condition will also receive suggestions how to monitor and self-regulate towards the goal (action planning, coping planning). Participants assigned to the Habit condition will also receive information on the importance of repetition of the same activity in the same context to develop habits.

The platform uses if-then algorithms to provide tailored feedback based on the participant’s responses. The feedback will be drawn from a database of messages incorporating feedback combinations tailored to the participant’s condition and responses to prompts for information on the website. The platform will store participants’ responses for each session, permitting tailoring of the intervention to the responses from the current session, as well as to responses from previously completed surveys. For instance, graphs displayed to the participants will include changes in physical activity throughout the intervention.

In addition, all groups, with the exception of the control group, will receive weekly text messages sent at the same time each week to provide further condition- and session-relevant feedback based on their most recently completed session and including a short summary of key points. There will be three introduction messages and each session has two session specific text messages, followed by a final concluding message. Participants will also receive regular fortnightly reminders to login to the intervention website when a new session is available sent via email. Up to three reminders will be sent per session (one every four days).

### Measures

Table 3 describes measures that will be taken during the study, specifying the type of outcome assessed, the measurement tool used, the number of questions included, and the time points when the measures will be administered.

**Table 3:** Measures taken at Time 1 and Time 2 and outcomes assessed

Measurement tool	Reference	Number of items		Outcome
		Time 1	Time 2	
		(baseline)	(3 months)	
Survey measures				
Confirmation of eligibility to participate in the study	N/A	8	-	Eligibility
Physical Activity Readiness Questionnaire (PAR-Q)	[45]	8	-	Readiness to undertake PA
Demographics	Commonly used items	14	6	Age, sex, marital status, ethnicity, education, weight, height, house income, postcode
International Physical Activity Questionnaire (short version)	[68, 69]	7	7	Physical Activity
Job-related physical activity	From: International Physical Activity Questionnaire (long version) [68]	8	8	Job-related physical activity
The Pittsburgh Sleep Quality Index (PSQI)	[70]	9	9	Sleep
Theory-relevant determinants of physical activity	Individual items taken from different questionnaires	30	30	Motivation, attitudes towards regular physical activity, outcome expectations, perceived behavioural control, subjective norms, intentions, barriers self-efficacy, action planning and coping planning, self-efficacy, goal facilitation and goal conflict
Self-report behavioural automaticity index	[77]	8	8	Behaviour automaticity
Personal Need for Structure Scale	[78]	12	-	Personal Need for Structure
Physical activity intentions	[72]	3	3	Physical Activity Intentions
Depression Anxiety Stress Scale 21 (DASS 21)	[79]	21	21	Depression, Anxiety, Stress
SF 12	[80]	12	12	Quality of life
Physical Activity Neighbourhood Environment Survey (PANES)	[81]	17	-	Perceptions of the environment in relation to PA
Internet use and access to the intervention	Items developed for this study	-	4	Internet use and access to the intervention
Internet self-efficacy scale	[83]	9	-	Internet self-efficacy
SUS and satisfaction	[82]	-	16	SUS and satisfaction
Physical advice acceptability	[66]	-	15	Physical advice acceptability
Delivery mode usability	[66]	-	5	Delivery mode usability
Usefulness questions	[66]	-	5	Intervention usefulness
Format-related questions	[66]	-	9	Format
Total items		158	158	
Outcome measures and moderators				
Physical activity objectively measured with GENEActiv	[47, 67]	✓	✓	Objectively measured physical activity
Sleep objectively measured with GENEActiv	[47]	Optional	Optional	Objectively measured sleep
Planning skill task	[90]	✓	-	Planning skills/ability

#### Physical activity

The primary outcome will be measured physical activity over a one-week period prior to the intervention (Time 1) and for a further week immediately post-intervention at the three-month follow-up occasion (Time 2) using accelerometers (GENEActiv Ltd.) [47, 67]. Participants will be asked to wear the unit continuously for the seven day period on the non-dominant upper arm to comply with hospital hygiene regulations. The GENEActiv device is water proof and participants will be informed that they are free to choose whether to wear it while swimming, showering, or sleeping. Data will be collected at 60 Hz epoch frequency. Step counts will be derived from the acceleration data using open-source macros and they will be used as proxy for physical activity [47]. Participants will be supplied with a band specifically designed to fasten the monitor to the upper arm.

Secondary outcomes will be assessed at baseline (Time 1) and post-intervention (Time 2), and will include subjective measures of physical activity, sitting time, sleep, depression, anxiety, stress, quality of life, theory-derived correlates of physical activity and self-reported weight. Demographics, perceived neighbourhood environment, personal need for structure, internet self-efficacy, and planning skills/ability will be assessed at baseline (Time 1) only. Secondary outcomes assessed only at the post-intervention stage (Time 2) will include measures of intervention accessibility, usefulness and satisfaction with the intervention, as well as acceptability of the advice provided. Participants’ website usage (e.g., time spent on pages with specific intervention content, number of website visits) will be assessed throughout the study using Google Analytics.

#### Physical activity (self-reported) and sitting time

Self-reported activity data will be assessed using a validated and reliable International Physical Activity Questionnaire (IPAQ) short version and work-related IPAQ module from the long version [68, 69].

#### Sleep

Sleep duration and quality will be measured using the valid and reliable Pittsburgh Sleep Quality Index [70]. We will also derive an objective measure of sleep using the GENEActiv activity monitors for study participants who choose to wear them during their sleep [71]. Total sleep time, number of awakenings, sleep onset and offset will be extracted from the device using open source macros [47].

#### Theory-derived psychological constructs

Psychological constructs derived from the motivation and volition theories and habit theory will be assessed using previously-validated and reliable psychometric questionnaires adapted to make reference to physical activity. The measures will include self-report measures of intentions [72], motivation, attitudes [73], outcome expectations [74], perceived behavioural control [73], subjective norms [73], barriers self-efficacy [75], action planning and coping planning [76], self-efficacy, goal facilitation and goal conflict [61]. Habit strength will be measured using the self-report behavioural automaticity index [63, 77]. Individual differences in tendencies to follow routines will be assessed using the personal need for structure scale [78].

#### Depression, anxiety and stress

Validated and reliable Depression, Anxiety and Stress Scale (DASS21) will be used to measure negative affective outcomes [79].

#### Quality of life

Quality of life will be assessed with the widely used, valid and reliable Medical Outcomes Survey - Short Form 12 (MOS-SF12) measure [80].

#### Perceived environmental factors

Participants ratings of the extent to which their home neighbourhood environment is supportive and conducive to physical activity will be assessed with the validated and reliable Physical Activity Neighbourhood Environment Survey (PANES) [81].

#### Internet use and intervention accessibility

Internet use and access will be measured with items developed specifically for this study. Usefulness and satisfaction with the intervention will be measured with the System Usability Scale [82]. Participants’ confidence in their ability to use the internet will be measured with validated and reliable internet self-efficacy scale [83].

### Characteristics of the intervention

Acceptability of the advice provided, intervention delivery and format will be measured with items used in previous studies [66].

### Demographics

Participants will be prompted to self-report a number of key demographic details: age, sex, marital status, ethnicity, education, weight, height, household income, postcode, the number of hours and days worked per week, work level (e.g., clinical nurse manager, registered nurse, patient care assistant), salary brackets associated with each employment level, type of work undertaken (e.g., shift-worker, non-shift worker, mix), and absenteeism in the last 3 months.

### Adverse effects

Participants will be encouraged to report any adverse effects they may experience during their participation in the intervention over the email or phone call.

### Sample size

Reviews of behavioural intervention studies have reported small to medium effects of web-delivered interventions aimed at increasing physical activity [84, 85]. Studies that excluded participants who are already active, which most closely represent the current protocol, reported a small-to-medium effect (*d* = 0.28) [84]. Therefore, to detect a similar effect on physical activity between intervention and control group, a total of 134 participants will be required to achieve 80% power with alpha set at *p* < .05. Based on an estimated drop out of 30% from web-based tailored interventions to increase physical activity [84], the final target sample size of 192 participants (48 per group) will be recruited at baseline. The study is powered to detect differences between the intervention group and control group (i.e., parallel 2-arm trial); not between the four study arms.

### Study hypotheses

1. We hypothesise significant increases in physical activity between baseline and 3-month follow-up in the three intervention conditions but no change in physical activity levels in the control group
2. We hypothesise that increases in physical activity at 3-month follow-up will be greatest in the habit intervention condition with smaller changes for the self-regulation and motivation conditions.
3. We hypothesise that participants allocated to the habit condition will have maintained the physical activity changes over time relative to the self-regulation, motivation, and control conditions at 3-month follow-up
4. We hypothesise that higher engagement in the program (e.g., frequency of logins, number of sessions completed, reported relevance and usefulness of the sessions content) will lead to greater change in physical activity at 3-month follow-up in all intervention groups.
5. We hypothesise that the effects of the habit condition on physical activity behaviour will be mediated by the self-reported habit development, effects of the self-regulation condition will be mediated by self-efficacy and planning, and the motivation condition will be mediated by the social cognitive variables of attitudes, motivation, and intention.

### Study analysis

The intervention will be evaluated following the recent guidance on process evaluation for complex interventions [86]. Data will be analysed using generalized linear mixed modelling (GLMM) [87] with a 4 (condition: control, motivation, self-regulation, habit) × 2 (time: baseline, 3-month follow-up) mixed-model design, accounting for nesting within hospital. Models will be conducting to compare change between conditions of the primary outcome (physical activity) and secondary outcomes (e.g., theory-derived psychological constructs, DASS21, MOS-SF12, and absenteeism). Mediation analyses will be conducted using path analytic models using Preacher and Hayes’ bootstrapped approach for multiple mediation [88]. Separate path models will be conducted for each condition. Each condition will be represented by a dichotomous dummy-coded variable (0 = did not receive intervention component, 1 = received intervention component). Each condition variable will be set as a predictor of physical activity at follow up with motivation and intention (motivation condition), perceived behavioural control and planning (self-regulation condition), and self-reported habit (habit condition) as multiple mediators in each analysis, respectively. Baseline physical activity will be included as a control variable. Intention-to-treat (baseline carried forward) will be utilised for missing data from drop-out. Other missing data will be imputed using multiple imputation with chained equations [89]. Both intention-to-treat and completers analyses will be reported.

## Discussion

Participation in regular physical activity shown to be related to improved health outcomes, reduced health care costs, and reduced disease risk (e.g., cardiovascular disease, Type 2 diabetes). Healthcare professionals including midwives, nurses and patient assistants are often involved in providing health-related behaviour change advice, yet their own health behaviour is relatively poor as demonstrated in occupational studies [7–9]. The *PATHS* study will examine the effectiveness of a web-based intervention evaluating different behavioural strategies to increase physical activity. The primary aim of the current trial is to test the main and additive effects of different theoretically-derived BCTs – motivational, self-regulatory and habit-based strategies – on physical activity and a range of secondary outcomes. We hypothesise that the habit condition, which includes the highest number of BCTs will be the most effective. However, we also acknowledge the potential for a larger number of BCTs may create problems for messages to be assimilated and recalled due to the relatively large amount of information provided. The greater information load may undermine, or even overturn, the effects of the BCTs on behaviour change. In addition, the current design does not allow for tests of interactions among sets of BCTs, rather the approach is focused on additive effects i.e. whether motivational interventions that include additional components (e.g., BCTs related to habit) are more effective compared to those that do not. However, this does not rule out the possibility of interactions among the different components affecting the result. Research aimed at identifying the optimum number and combination of BCTs is required to inform the design of future complex interventions.

In addition, the current study will focus on testing theoretically-distinct groups of techniques and their additive effects on health behavior change. The findings of the study will enable us to evaluate the potential mechanisms by which BCTs derived from motivation, volition and habit theories affect change in health-related behaviour. Our research will also enable us to test the mediators of the intervention effects to increase participants’ physical activity and quality of life, and to reduce work-related stress and absenteeism. We will be able to do this by comparing the effects of each intervention component (e.g., self-regulation) on outcomes mediated by specific theory-based psychological constructs (e.g., planning, self-efficacy) conceptually related to the component.

Personal characteristics of the intervention participants will be assessed to determine for whom the different intervention content is most suitable. Potential moderators of intervention effects will include level of engagement in the program and planning skills. Determining the characteristics of potential users will enable the future matching of participants with the most suitable intervention content. In addition, by providing opportunity for participants to report on the usability of the platform in healthcare professionals, we will be able to ascertain the extent to which the online intervention is feasible in this population and whether it would likely be used by the target population if it were rolled-out on a large scale.

### Implications

The study described here has important implications for public health practitioners and researchers designing behaviour change interventions. The intervention will make a unique contribution to physical activity promotion and health research by testing the effectiveness of a computer-tailored web-based intervention in promoting physical activity and stress reduction in hospital workers; an under-researched group at risk of high stress. In addition, the intervention will add to theory and knowledge on behaviour change in health contexts by testing whether the inclusions of self-regulatory and habit-forming BCTs are more effective in promoting behaviour change and health-related outcomes relative to BCTs targeting motivation alone and an information only control. This is important as it will assist in elucidating the ‘active ingredients’ of the intervention and the mechanisms by which they exert their effects. The current research will also assist in identifying the key mediators of intervention effects, providing data on the mechanisms by which each intervention condition affects changes in physical activity behaviour.

## Abbreviations

BCT: Behaviour change technique
DASS21: Depression Anxiety and Stress Scale 21
IPAQ: International Physical Activity Questionnaire
PANES: Physical Activity Neighbourhood Environmental Scale
PARQ: Physical Activity Readiness Questionnaire
PATHS: Physical Activity Tailored intervention in Hospital Staff
RCT: Randomised controlled trial
SF12: Short Form 12 (Quality of Life Measure)
SMS: Short message service
SUS: The System Usability Scale
TPB: Theory of Planned Behaviour
